# Broadband absorber with dispersive metamaterials

**DOI:** 10.1515/nanoph-2022-0777

**Published:** 2023-03-22

**Authors:** WonHeum Han, Q-Han Park

**Affiliations:** Physics, Korea University, Seoul, Korea

**Keywords:** broadband absorber, electromagnetic wave absorbers, equivalent circuit model, Lorentz model, metamaterial, perfect electric conductor

## Abstract

A broadband absorber that utilizes a dispersive metamaterial and covers the entire microwave X-band (8–12 GHz) is proposed in the present study. An ideal absorber attached to the surface of a perfect electric conductor requires the permittivity of the absorbing layer to be anomalously dispersive in the targeted broad frequency band. We show that anomalous dispersion of the permittivity for the X-band can be fitted to a two-pole Lorentz oscillator model and realized with the use of a double-layered, square-loop metamaterial. We explain the connection between the two-pole oscillator model and the double-layered, square-loop metamaterial using an equivalent circuit model and present explicit design rules for the metamaterial. We fabricate a 4-mm-thick metamaterial absorber with flexible silicon rubber, a resistor element, and conductive wire using carbon and silver conductive ink. Our metamaterial absorber achieves a reflectance of less than −20 dB over the entire X-band region.

## 1 Introduction

Minimizing reflection is an important objective in stealth technology, city construction, and automobile electronic components in order to avoid unwanted interference from electromagnetic (EM) waves. In this context, the use of an absorbing layer on a metal surface to absorb EM waves is one of the most efficient methods for the suppression of reflection. Various types of absorbing layer have been proposed to target the microwave X-band, including homogeneous monolayers [1, 2], multilayered heterogeneous materials [3, 4], and structured metamaterial layers [5, 6].

Single-layer absorbers positioned on a metal surface with a gap of the Salisbury screen [1, 2] exhibit a sharp reduction in the reflection spectrum at the target frequency, meaning they are not suitable for operation over a broad frequency band, while the requirement that the spacing layer has a thickness of one-quarter of the target wavelength inevitably increases the device size and complexity [7]. Multilayered absorbers are fabricated by stacking dielectric and carbon-based absorbing materials on a metal surface [3, 4], thus broadening the absorption frequency band. The material and structural parameters of a multilayered architecture can be optimized to target a broader operational range via trial-and-error, but this approach suffers from constraints associated with material availability, error tolerance, and the fabrication process. To overcome these limitations, metamaterials have been utilized in the form of structured layers. However, despite these efforts, an average reflectance below −20 dB within the microwave X-band (8–12 GHz) has yet to be achieved [8–14].

In the present study, we adopt a different approach to the problem. We determine the conditions required for an ideal absorber to completely absorb monochromatic EM waves and demonstrate that these conditions can be met using a simple design based on a Lorentz oscillator model. To ensure operation over a broad frequency band, the dielectric constant of the absorbing layer should be anomalously dispersive. We show that this anomalous dispersion [15, 16] is in accordance with a two-pole Lorentz oscillator model, which can be effectively realized with the use of a double-layered, square-loop metamaterial. This is illustrated using an equivalent circuit model for the metamaterial. We also present an explicit design for a double-layered, square-loop metamaterial as an X-band absorber. We fabricate this metamaterial with flexible silicon rubber, a resistor element, and conductive wire using carbon and silver conductive ink and find that the effective dielectric constant is in good agreement with the required anomalous dispersion. Of particular note is that our proposed metamaterial absorber achieves a reflectance of under −20 dB over the entire microwave X-band.

## 2 Broadband absorber

### 2.1 Ideal absorbing layer

**Figure 1:**
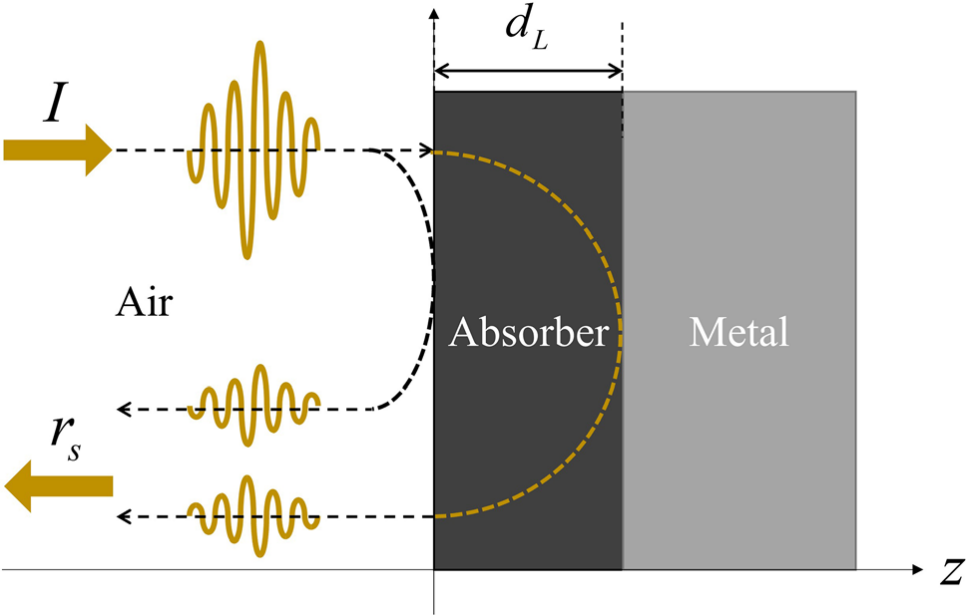
Light incident from the air to a unit cell consisting of an ideal absorber on a perfect electric conductor (PEC).

Consider an EM wave from the air incident to the planar interface of an absorber attached to a metal surface (Figure 1). For simplicity, we only consider normal incidence and assume that the metal is a perfect electric conductor (PEC) and that the absorber (thickness *d*_*L*_) has the complex refractive index $\sqrt{\varepsilon_{L}}=n+i\kappa$ . For a monochromatic wave of wavelength *λ* incident from the air, the reflected wave arises from the mismatched impedance with the reflection coefficient *r*:

(1) $$\begin{array}{c} r=\frac{Z_{L}\left(e^{\text{i}kd_{L}}-e^{-ikd_{L}}\right)-Z_{0}\left(e^{\text{i}kd_{L}}+e^{-ikd_{L}}\right)}{Z_{L}\left(e^{\text{i}kd_{L}}-e^{-ikd_{L}}\right)+Z_{0}\left(e^{\text{i}kd_{L}}+e^{-ikd_{L}}\right)}\\ k_{0}=\frac{2\pi }{\lambda },\;k^{L}=\left(n+i\kappa \right)k_{0},\;Z_{0}=\sqrt{\frac{\mu_{0}}{\varepsilon_{0}}},\;Z_{L}=\frac{1}{n+i\kappa }\sqrt{\frac{\mu_{0}}{\varepsilon_{0}}} \end{array}$$

where *k*_0_, *Z*_0_(*k* ^*L*^, *Z* ^*L*^) are the wavenumber and impedance, respectively, in a vacuum and in the layer. Complete absorption occurs when *r* is zero, for which the refractive index satisfies

(2) $$e^{2i\left(n+i\kappa \right)k_{0}d_{L}}=\frac{1-n-i\kappa }{1+n+i\kappa }=e^{2i\left[\left(n+i\kappa \right)k_{0}d_{L}+m\pi \right]},\;m=0,1,2,\ldots$$

**Figure 2:**
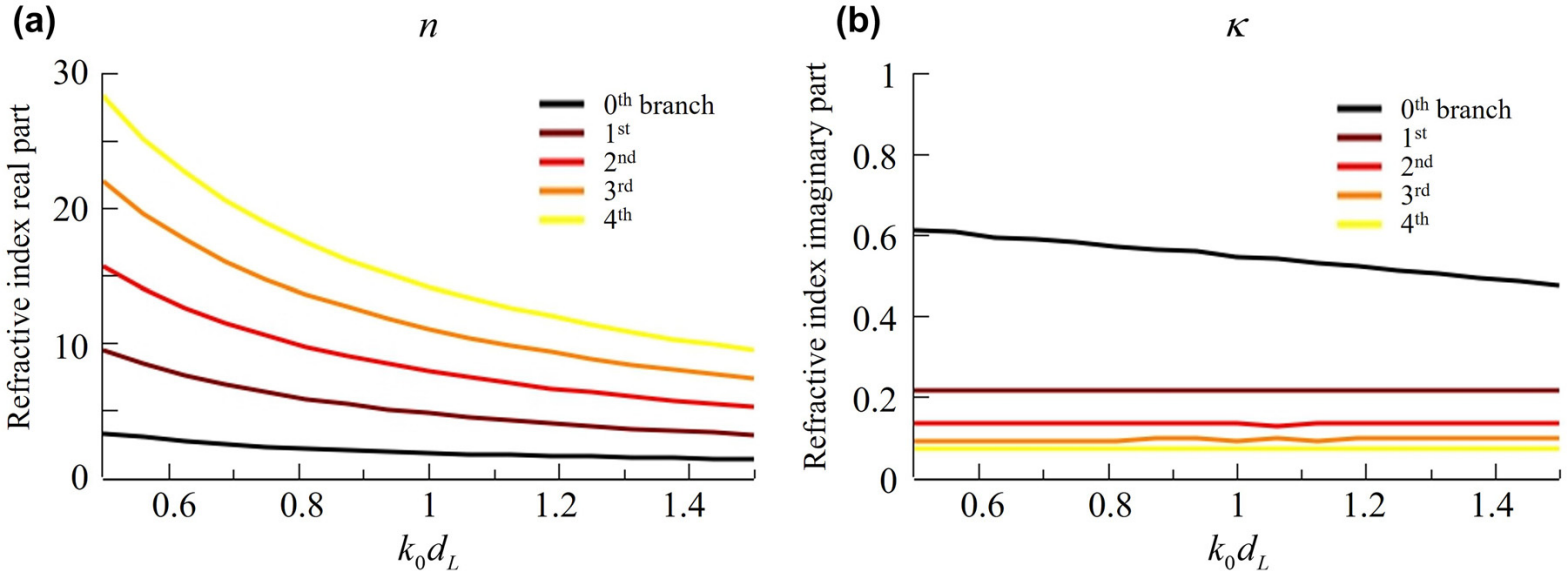
Dispersion of the refractive index branches for an ideal absorber: (a) real and (b) imaginary parts of the refractive index.

Since equation (2) is complex with the complex refractive index *n* + *ik*, we separate the real and imaginary parts of the equation and solve them for *n*, *k*. We note that *n*, *k* are functions of the dimensionless quantity *k*_0_ *d*_*L*_ up to integer -*m* branches. Although analytic solutions are not available, we can solve this numerically within the region of interest for each branch. The results in Figure 2 show that index *n* is anomalously dispersive, i.e., it reduces as the frequency increases. Index *n* grows for higher branch number *m* because it supports more nodes inside the layer. From this point, we restrict our focus to the lowest branch (*m* = 0).

### 2.2 Anomalous dispersion and oscillator fitting

**Figure 3:**
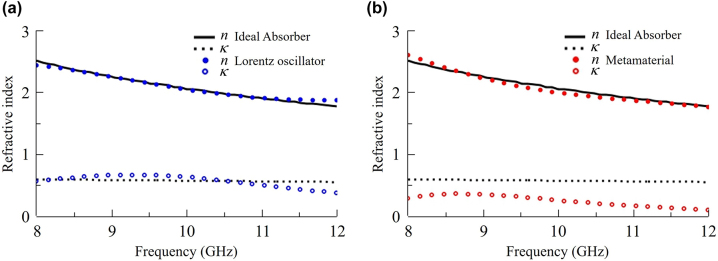
Anomalous refractive index for an ideal X-band absorber of 4 mm thickness with (a) Lorentz model fitting and (b) metamaterial fitting.

Anomalous dispersion is not commonly observed in natural materials, arising only within a narrow frequency band near resonance and accompanied by strong absorption. Within the region of interest, such as the X-band in Figure 3(a), anomalous dispersion can be modeled using a multipole Lorentz oscillator model of a dielectric constant that satisfies the physical causality constraint. In our case, we fit the numerically calculated dispersion with a two-pole Lorentz oscillator model by adjusting the parameters of the Lorentz oscillators as follows:

(3) $$\varepsilon_{\text{Lorentz}}\left(f\right)=\varepsilon_{\inf}+\underset{j=1,2}{\sum }\frac{\omega_{pj}^{2}}{\omega_{0j}^{2}-4\pi^{2}f^{2}-2\pi i\Gamma_{j}f}$$

were *ɛ*_inf_ is the relative permittivity for the large frequency limit, and *ω*_*p*_, *ω*_*o*_, and Γ are the plasma frequency, resonance frequency, and damping factor, respectively. The fitting parameters for various thicknesses of the absorber are summarized in Table 1.

**Table 1:** Fitting parameters for the two-pole Lorentz oscillator model.

	Thickness of the absorber	4 mm	5 mm	6 mm	7 mm	8 mm
Pole 1 (GHz)	Resonance frequency [angular]	350.6	458.8	539.6	670.8	888.8
	Plasma frequency	616.9	616.7	616.6	616.7	616.6
	Damping factor	31.1	31.1	31.1	31.1	31.1
Pole 2 (GHz)	Resonance frequency [angular]	60.3	60.2	56.9	56.0	55.9
	Plasma frequency	74.4	72.8	71.6	70.6	70.1
	Damping factor	34.1	42.2	48.3	55.3	66.0
*ɛ*_inf_		1	1	1	1	1

**Figure 4:**
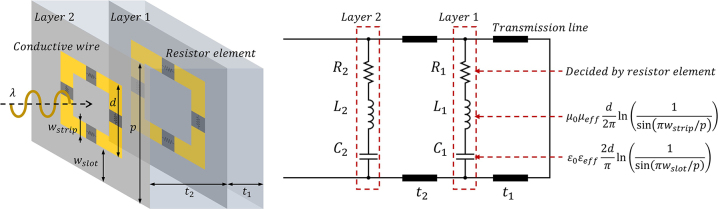
Equivalent circuit diagram for a double-layer metamaterial.

To realize the permittivity of a two-pole Lorentz oscillator, we consider a double-layered, square-loop metamaterial. Square-loop metamaterials exhibit resonance features that can be effectively described using an equivalent circuit model [5]. Our double-layered metamaterial is also described using an RLC parallel circuit and lumped elements [17] in Figure 4. These lumped elements are related to the structural parameters of the metamaterial, in particular the period of the unit cell *p*, the width of the square loop strip *w*_strip_, and the slot spacing between the square loops. Even though the lumped element circuit model describes the transmission line accurately, it agrees with the propagation properties of the metamaterial as an effective continuous medium only for the limit *λ* ≫ *p* ≫ *w*_strip_. Here, we only note the appearance of two distinct resonances in the double-layered, square-loop metamaterial but do not pursue this further. Rather, instead of relying on a circuit-model–based approach, we design a metamaterial via a direct optimization process, as described in the next section.

To confirm the validity of our direct optimization process, we use the Nicolson–Ross–Weir (NRW) parameter retrieval method [18, 19] to obtain the effective refractive index for the designed double-layered, square-loop metamaterial. A comparison between the dispersion of an ideal absorber and the NRW-retrieved dispersion of the metamaterial is presented in Figure 3(b).

### 2.3 Design of the double-layered, square-loop metamaterial

**Figure 5:**
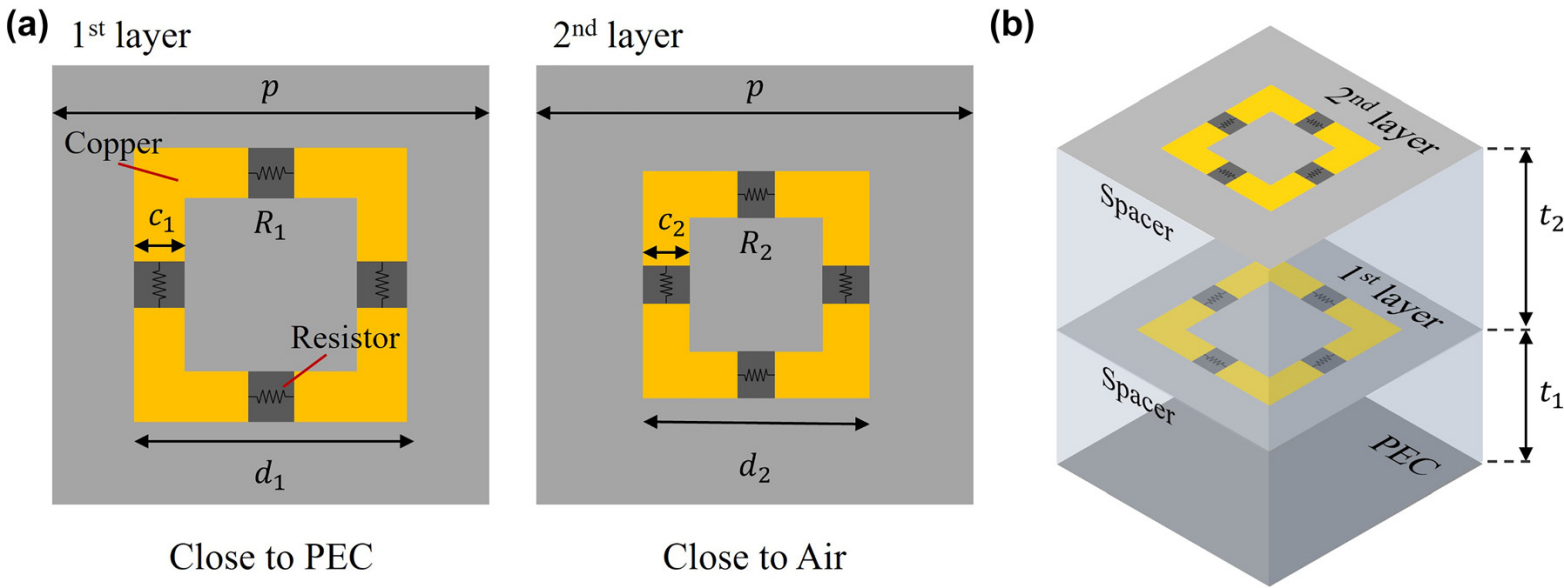
Schematic diagrams of (a) the unit cell and (b) the two metamaterial layers on a PEC.

The metamaterial absorber consists of two layers of a copper square loop attached to a silicon rubber substrate (Figure 5). To balance damping, the copper square loop is divided into four pieces that are connected by resistor elements. Using the particle swarm algorithm, we optimize the structural parameters to minimize the average reflectance over the X-band range. Using copper (which is assumed to be a PEC) and silicon rubber (dielectric constant = 3.2), we optimize the structural parameters for layer 1 (the PEC side) and layer 2 (the air side) in terms of the unit cell size (*p*_1_ = *p*_2_ = 14.4 mm), the side of the copper square (*d*_1_ = 7.3 mm *d*_2_ = 6.8 mm), the width of the copper loop (*c*_1_ = 1.3 mm *c*_2_ = 0.7 mm), and the thickness of the rubber substrate (*t*_1_ = 1.5 mm *t*_2_ = 2.5 mm). The frequency range is 8–12 GHz. For the resistor, the lumped elements built into the simulation program CST Studio Suite are selected. Simulations are performed with three resistor pairs: set 1 (*R*_1_ = 40 Ω *R*_2_ = 150 Ω), set 2 (*R*_1_ = 30 Ω *R*_2_ = 155 Ω), and set 3 (*R*_1_ = 45 Ω *R*_2_ = 140 Ω). The unit cell size (*p*_1_, *p*_2_) of metaatom is relatively big compare to the wavelength (30 *mm*) of center frequency (10 *GHz*). However, it is still within the range where reflection and absorption are governed by the zeroth order of diffraction and the effective refractive index description works. The results are summarized in Figure 9.

## 3 Experiment

### 3.1 Sample fabrication

**Figure 6:**
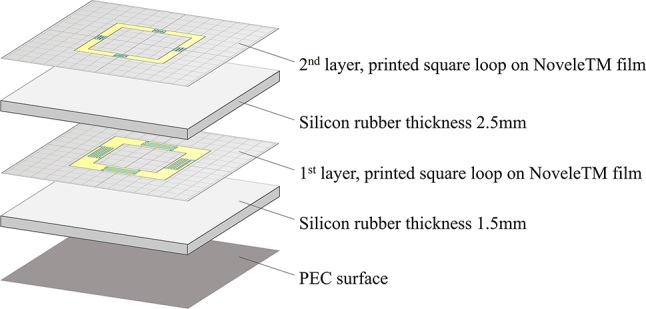
Design of the unit cell for the experimental samples.

**Figure 7:**
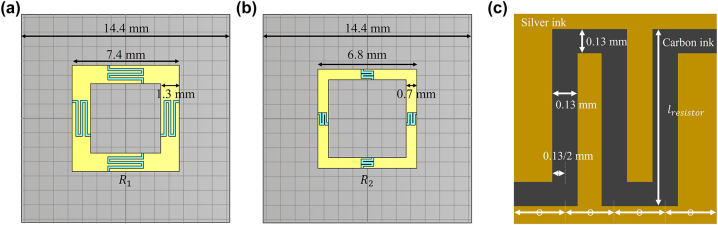
Design of the unit cell for the experimental samples: (a) circuit layer close to the PEC, (b) circuit layer close to the air, and (c) resistor unit design. The resistance is adjustable depending on length *l*_resistor_.

The double-layered metamaterial is prepared so that each layer contains 13 × 13 unit elements. The layers are fabricated using conductive ink, printing film from Nova Centrix, and a silicon rubber substrate. The overall size of the absorber is 187.2 mm × 187.2 mm × 4 mm. Details of the unit elements are presented in Figures 6 and 7. The resistor elements and conducting wires in the unit elements are fabricated from conductive silver and carbon ink. The thickness of the silicon rubber (refractive index = 1.8), substrate between the PEC and the first layer is 1.5 mm and that between the first and second layers is 2.5 mm. We discover that a reasonable resistance lies between 30 Ω and 155 Ω. We control the resistance by adjusting the ratio of silver to carbon ink and changing the length of the resistor units. The shape of the resistor units is presented in Figure 7(b). By changing the length of the resistor units (*l*_resistor_) from 0.8 mm to 2.5 mm, the resistance can be adjusted from 30 Ω to 180 Ω.

### 3.2 Measurement

**Figure 8:**
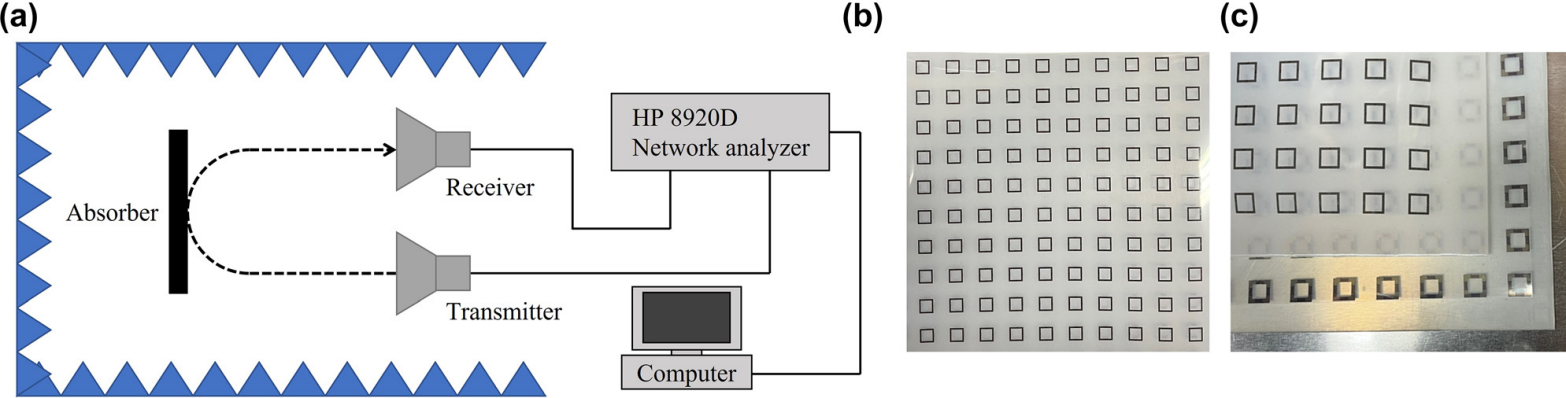
Experiment setting. (a) Measurement setup. (b) Metamaterial absorber sample. (c) Metamaterial absorber sample.

**Figure 9:**
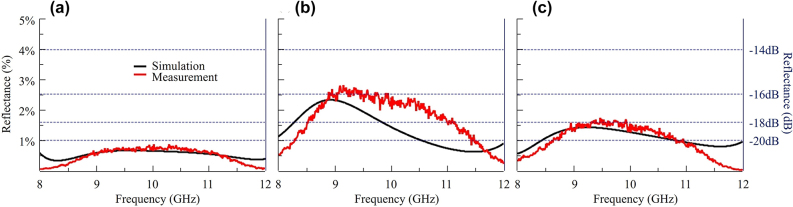
Comparison of simulation and measurement results for reflectance with different resistor designs: (a) *R*_1_ 40 Ω, *R*_2_ 150 Ω, (b) *R*_1_ 30 Ω, *R*_2_ 155 Ω, and (c) *R*_1_ 35 Ω, *R*_2_ 140 Ω.

A schematic diagram of the setup used to measure the reflectance (*R*) is presented in Figure 8(a). The system consists of a pair of X-band horn antennas, a sample holder, an anechoic chamber, an HP 8920D network analyzer, and a computer for S-parameter data acquisition. The reflectance is calculated from the S-parameters after calibration. The S-parameters of the chamber background and a metal plate of the same size as the metamaterial sample are measured. The reflection signal of the metamaterial and the metal plate excluding the reflection signal of the chamber background are used for the calibration. The frequency range between 8 and 12 GHz is scanned, and the measurement results are presented in Figure 9.

## 4 Results and discussion

We fabricate samples and measure the reflectance for three types of resistor element that are selected from the optimization process. Figure 9 presents the measured reflectance and that predicted from the simulation. The best absorbing performance is achieved by the resistor pair *R*_1_ = 40 Ω, *R*_2_ = 150 Ω, which produce a reflectance of under −20 dB over the entire X-band region, while an average reflectance within the X-band of about −23 dB. The performance of our proposed double-layered metamaterial is compared in terms of its maximum and average reflectance, polarization coverage, thickness of the absorber, number of layer materials, and flexibility with that of other previously reported absorbers in Table 2. The average reflectance of the other absorbers in the target X-band ranges between −12 dB and approximately −20 dB [1, 3], [4], [5, 8–14], which is significantly higher than the −23 dB achieved by our metamaterial absorber. Because we use a rubber substrate, our absorber is also flexible, potentially increasing the range of applications to which it could be applied.

**Table 2:** Comparison of square-loop periodic models.

	R (max)	R (max)	R (avg)	R (avg)	TE/TM cover	Thickness	Number of	Flexibility
	Simulation	Measurement	Simulation	Measurement			materials	
Single layer [1]	−10.9 dB	−10.8 dB	−11.8 dB	−11.7 dB	⚬	2 mm	1–2	×
Multilayer [3]	No data	−9.0 dB	No data	−12.7 dB	⚬	No information	3–5	×
Multilayer [4]	−10.6 dB	No data	−18.8 dB	No data	⚬	2 mm	1–4	×
Metamaterial [5]	−18.5 dB	−22.5 dB	−19.3 dB	−27.0 dB	⚬	7–8 mm	2	×
Hybrid [8]	−8.2 dB	−7.9 dB	−17.3 dB	−16.5 dB	⚬	3–7 mm	2–4	×
Our work	−21.6 dB	−20.6 dB	−22.7 dB	−23.1 dB	⚬	4 mm	3	⚬

In this work, we presented a prototype metamaterial absorber utilizing the resonance of a square-loop geometry and the anomalous dispersion resulting from an asymmetric double-layered structure. We show that anomalous dispersion is critical for the broadband operation of an absorber, and this can be achieved physically following the fitting of a Lorentz model for permittivity. We also present design rules for the metamaterial so that two-pole Lorentz oscillators can be successfully realized. Experimental measurements confirm the validity of our approach, highlighting the remarkable performance of our absorber. In addition, because it represents a systematic method that is not confined to the X-band, other applications can potentially utilize our approach.
